# Closed Thoracic Trauma as an Exceptional Cause of Pneumorrhachis: A Case Report

**DOI:** 10.7759/cureus.59437

**Published:** 2024-05-01

**Authors:** Chirwa Abdillahi Mahamoud, Najwa Benslima, Abderrahim Bourial, Naima El Benna, Amal Rami

**Affiliations:** 1 Radiology, Cheikh Khalifa International University Hospital, Mohammed VI University of Sciences and Health, Casablanca, MAR; 2 Radiology, Mohammed VI International University Hospital, Mohammed VI University of Sciences and Health, Casablanca, MAR; 3 Otolaryngology, Cheikh Khalifa International University Hospital, Mohammed VI University of Sciences and Health, Casablanca, MAR; 4 Radiology, Centre Hospitalier Universitaire Ibn Rochd Hôpital 20 Août, Casablanca, MAR

**Keywords:** tomodensitometry, thoracic injuries, pneumothorax, aerorachia, pneumorrhachis

## Abstract

Pneumorrhachis, a rare clinical entity, refers to the presence of air in the spinal canal. Air can enter the spinal canal through various pathways, including the lungs and mediastinum (the space between the lungs), or directly from external sources due to trauma or infection. In rare cases, pneumorrhachis may result from repeated secondary Valsalva maneuvers, which is a complication of large-area pneumothorax.

In this case report, we discuss a 36-year-old male patient who was involved in a high-intensity road accident. The injury assessment revealed significant findings including a large left pneumothorax, a right pneumothorax, multiple rib fractures, and the presence of pneumorrhachis. The entry of air into the spinal canal originated from the pleural space, likely through injuries to the parietal pleura.

Rarely reported, closed thoracic trauma is an exceptional cause of pneumorrhachis. This unique mechanism of injury has been described in a limited number of publications addressing traumatic pneumorrhachis. The identification of pneumorrhachis in a traumatized patient should prompt further investigation to explore other potential injuries that may elucidate the formation of this intraspinal gas collection.

## Introduction

Pneumorrhachis refers to the presence of air in the spinal canal, and it can lead to an array of symptoms of different gravity. Air can enter the spinal canal through various pathways, including the lungs and mediastinum (the space between the lungs), or directly from external sources due to trauma or infection [1]. In rare cases, pneumorrhachis may result from repeated secondary Valsalva maneuvers, which is a complication of large-area pneumothorax [2]. The abnormal presence of air within the spinal canal can lead to compression or displacement of the spinal cord and nerves, resulting in neurological symptoms [3]. Pneumorrhachis can be classified into two primary types based on the location of the air: intradural pneumorrhachis and extradural pneumorrhachis [4]. Diagnosing typically involves imaging studies such as X-rays, computed tomography (CT), or magnetic resonance imaging (MRI) [5].

Pneumorrhachis is relatively rare, and its clinical significance depends on the underlying cause, the amount of air present, and the location within the spinal canal. While traumatic pneumorrhachis often remains asymptomatic and may not necessitate immediate treatment, it is crucial to recognize its presence as an indicator of severe trauma [6]. Identifying pneumorrhachis necessitates careful patient monitoring due to the underlying severity of the trauma [7]. In this report, we present the case of a male patient who presented with pneumorrhachis after a road traffic accident in which she experienced multiple fractures and injuries leading to large-volume pneumothorax and subcutaneous emphysema.

## Case presentation

A 36-year-old male patient, with no pertinent medical or surgical history, was admitted to the emergency department due to polytrauma resulting from a motor vehicle accident. Upon admission, his Glasgow Coma Scale was 15/15. Hemodynamic vitals were within normal limits, but his oxygen saturation level was recorded at 94%. The patient presented with craniofacial, thoracic, and abdominal injuries. No neurological deficits were evident, and there was no initial loss of consciousness or vomiting. The patient reported dyspnea, chest pain, and upper back pain. Clinical examination revealed a deep facial wound, left thoracic deformity, exacerbated dyspnea, and subcutaneous emphysema.

**Figure 1:**
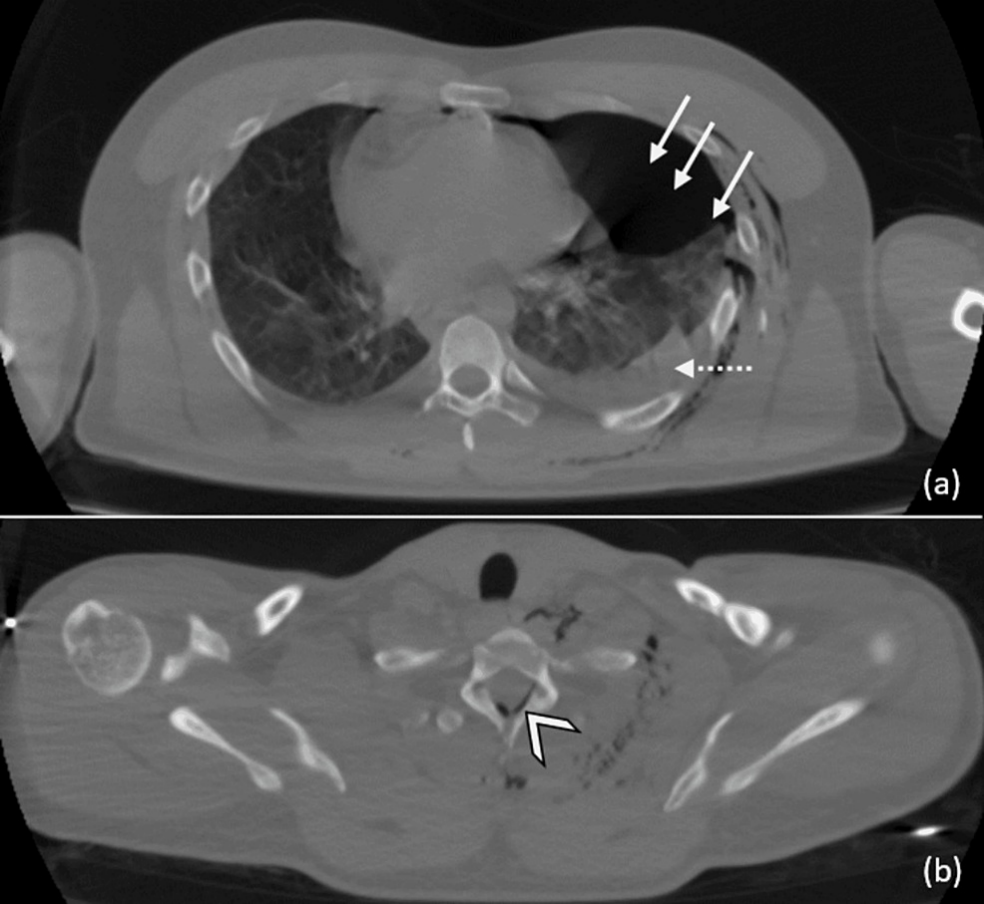
(a) Axial non-contrast chest CT images in the lung window, showing a left pneumothorax (solid white arrows) and minimal left-sided pleural effusion (dashed white arrow). (b) Presence of air within the spinal canal (white arrowhead). CT: computed tomography

**Figure 2:**
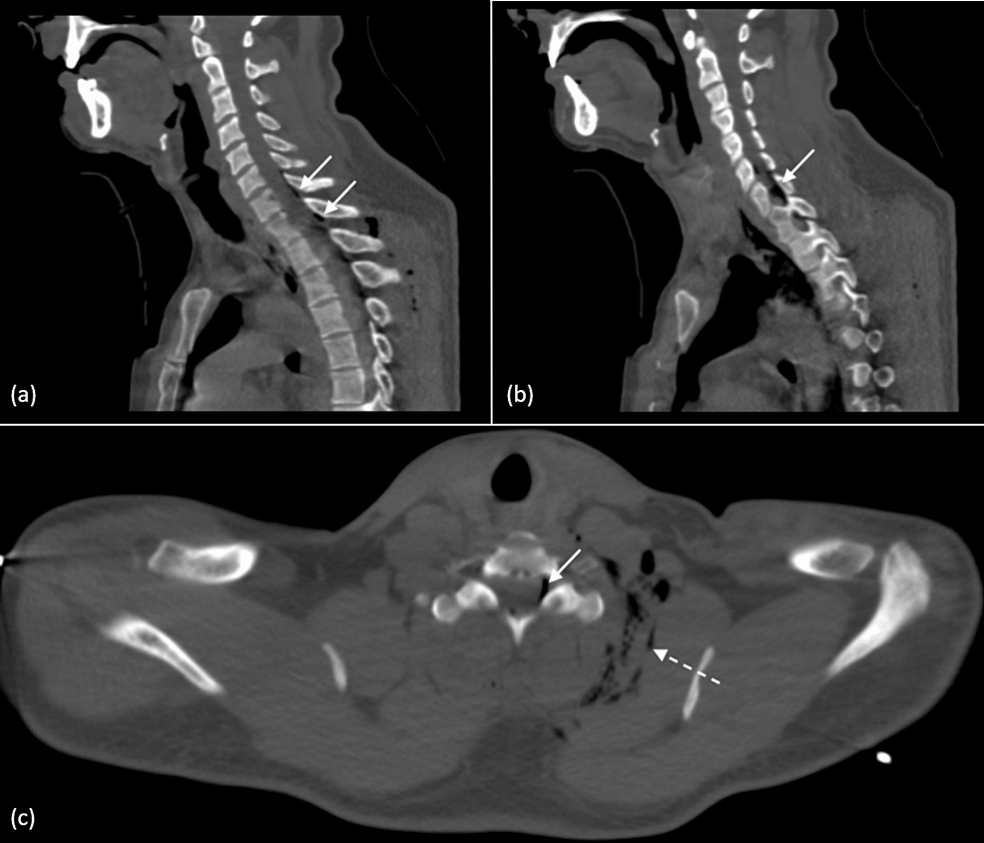
(a-b) Non-contrast thoracic CT sagittal images in bone window setting, displaying epidural pneumorrhachis (solid white arrow). (c) Axial cut in bone window setting, displaying left cervical subcutaneous emphysema (dashed white arrow) and epidural pneumorrhachis (solid white arrow). CT: computed tomography

A comprehensive whole-body CT scan was conducted following the standard protocol, including scans with and without injection of contrast. The imaging results disclosed several significant findings, including a large left pneumothorax with pleural effusion, a small right pneumothorax, subcutaneous emphysema in the cervical-thoracic and dorsal regions (Figure 1a, Figure 2c), and the presence of air within the spinal canal at the T7 and T8 levels (pneumorrhachis) (Figure 1b, Figure 2a, 2b).

Furthermore, multiple displaced left rib fractures were observed by body CT scan, spanning from K1 to K9, with no evidence of vertebral fractures. The patient underwent multidisciplinary treatment for the aforementioned conditions. A decision was made to employ a straightforward surveillance strategy for the pneumorrhachis, and it was carefully monitored.

## Discussion

Gordon et al. [8] initially reported the rare occurrence of air surrounding the dura mater spinalis in 1977. Various terms have been used to denote this phenomenon in the literature, including intraspinal "pneumocele" or "pneumocoele," "spinal and epidural emphysema," and "aerorachia" [9].

Pneumorrhachis can arise from a variety of causes, including traumatic incidents, pulmonary diseases, infections, barotrauma, and recent iatrogenic interventions (surgical, lumbar punction, and invasive diagnostic procedures) [2,8]. In rare cases, pneumorrhachis may be associated with conditions such as pneumothorax [10], pneumomediastinum, or pneumoperitoneum [9]. Pneumorrhachis may also occur spontaneously without any identifiable precipitating cause [9,11]. Pneumorrhachis represents a rare complication associated with pneumothorax, with the ingress of air into the spinal canal likely occurring from the pleural space, possibly through lesions in the parietal pleura and subsequently into the epidural or subarachnoid spaces via the neural foramina [9,10]. The accumulation of air tends to occur within the posterior epidural space due to its relatively lower resistance in comparison with the anterior epidural space, which harbors a more densely populated vascular network [11,12].

The potential mechanism of pneumorrhachis in our patient could be attributable to the entry of air into the epidural space because of the rupture of the posterior parietal pleura due to bilateral pneumothorax, which was particularly pronounced on the left side, accompanied by subcutaneous emphysema. The diagnosis of pneumorrhachis is frequently incidental, with detection occurring as a result of CT imaging during the investigation of significant thoracic or spinal lesions [8]. CT represents the gold standard for accurate diagnosis. Other imaging modalities such as X-rays or MRI may also prove beneficial in diagnosing pneumorrhachis [4,11].

Treatment approaches hinge upon the underlying etiology and the presenting symptoms [6]. Asymptomatic cases do not necessitate active intervention and can be managed conservatively. Conversely, in severe manifestations of spinal cord compression, surgical intervention may be necessary [8]. A tailored approach, based on the specific clinical scenario, guides the appropriate management strategy for patients presenting with pneumorrhachis.

## Conclusions

Pneumorrhachis is an infrequent phenomenon, often incidentally detected through imaging. Nevertheless, its occurrence commonly indicates the presence of significant underlying pathologies, necessitating a heightened level of awareness. While often asymptomatic and transient, subarachnoid pneumorrhachis can manifest with neurological impairments, necessitating surgical intervention. In our case, the heightened intrathoracic pressure after pneumothorax emerged as the most plausible etiology.
